# Rectal Swab–based Targeted Prophylactic Antibiotics Reduce Infectious Complications After Transrectal Prostate Biopsy: A Systematic Review and Meta-analysis of Randomized Controlled Trials

**DOI:** 10.1016/j.euros.2025.08.007

**Published:** 2025-09-11

**Authors:** Ichiro Tsuboi, Mehdi Kardoust Parizi, Akihiro Matsukawa, Marcin Miszczyk, Tamás Fazekas, Angelo Cormio, Tatsushi Kawada, Satoshi Katayama, Takehiro Iwata, Kensuke Bekku, Koichiro Wada, Pierre I. Karakiewicz, Piotr Chlosta, Alberto Briganti, Motoo Araki, Shahrokh F. Shariat

**Affiliations:** a Department of Urology, Comprehensive Cancer Center, Medical University of Vienna, Vienna, Austria; b Department of Urology, Faculty of Medicine, Shimane University, Shimane, Japan; c Department of Urology, Okayama University Graduate School of Medicine, Dentistry and Pharmaceutical Sciences, Okayama, Japan; d Department of Urology, Shariati Hospital, Tehran University of Medical Sciences, Tehran, Iran; e Department of Urology, Jikei University School of Medicine, Tokyo, Japan; f Collegium Medicum - Faculty of Medicine, WSB University, Dąbrowa Górnicza, Poland; g Department of Urology, Semmelweis University, Budapest, Hungary; h Department of Urology, Azienda Ospedaliero-Universitaria Ospedali Riuniti Di Ancona, Università Politecnica Delle Marche, Ancona, Italy; i Cancer Prognostics and Health Outcomes Unit, University of Montreal Health Centre, Montreal, Quebec, Canada; j Department of Urology, Jagiellonian University Medical College, Krakow, Poland; k Unit of Urology/Division of Oncology, Gianfranco Soldera Prostate Cancer Lab, IRCCS San Raffaele Scientific Institute, Milan, Italy; l Vita-Salute San Raffaele University, Milan, Italy; m Department of Urology, University of Texas Southwestern Medical Center, Dallas, TX, USA; n Department of Urology, Weill Cornell Medical College, New York, NY, USA; o Department of Urology, Second Faculty of Medicine, Charles University, Prague, Czechia; p Division of Urology, Department of Special Surgery, The University of Jordan, Amman, Jordan; q Karl Landsteiner Institute of Urology and Andrology, Vienna, Austria; r Department of Urology, Semmelweis University, Budapest Hungary; s Research Center for Evidence Medicine, Urology Department, Tabriz University of Medical Sciences, Tabriz, Iran

**Keywords:** Febrile urinary tract infection, Targeted prophylactic antibiotics, Transrectal prostate biopsy, Sepsis

## Abstract

**Background and objective:**

Transperineal ultrasound-guided prostate biopsy is the recommended approach in guidelines, while transrectal ultrasound-guided prostate biopsy (TRUS-PB) is still widely used to diagnose prostate cancer (PCa); however, it is associated with a significant rate of infectious complications. We aimed to assess the efficacy of targeted prophylactic antibiotics (TPAs), based on rectal swabs, in reducing the incidence of infectious complications after TRUS-PB compared with empiric prophylactic antibiotics.

**Methods:**

PubMed, Web of Science, and Scopus were queried in December 2024 for randomized controlled trials (RCTs) comparing infectious complications between patients who received TPAs based on rectal swab culture before TRUS-PB and those who received empiric prophylactic antibiotics before TRUS-PB (PROSPERO: CRD42024523794). The primary outcomes were the incidence rates of febrile urinary tract infection (fUTI) and sepsis.

**Key findings and limitations:**

Overall, nine RCTs (*n* = 3002) were included in our analyses. The incidence of fUTI was approximately half as high in patients who received TPAs as in those who received empiric prophylactic antibiotics (*n* = 3002, 2.7% vs 5.2%, risk ratio [RR]: 0.54, 95% confidence interval [CI]: 0.36–0.81, *p* = 0.003). Based on these pooled incidence rates, the number of patients needed to treat to prevent fUTI after TRUS-PB was 40; however, there was no statistically significant difference in the incidence of sepsis between patients receiving TPAs and those who received empiric antibiotic prophylaxis (*n* = 2735, 1.3% vs 1.8%, RR: 0.74, 95% CI: 0.31–1.75, *p* = 0.4).

**Conclusions and clinical implications:**

TPAs based on rectal swab culture significantly reduces the incidence of fUTI in patients who undergo TRUS-PB for PCa diagnosis compared with that in patients who receive empiric prophylactic antibiotics; however, there is insufficient evidence to assess its effect on the risk of sepsis. We recommend, based on the clinically relevant reduction in the incidence of fUTI, performing rectal swab–based TPAs in patients undergoing TRUS-PB.

**Patient summary:**

We reviewed infections occurring after transrectal prostate biopsy in over 3000 patients. The use of antibiotics chosen based on a simple rectal swab decreased the rate of postbiopsy fever and urinary tract infections by half compared with the use of standard antibiotics. More research is needed to understand whether this approach also prevents the rare but serious complication of sepsis.

## Take Home Message

Rectal swab–based targeted prophylactic antibiotics halve the febrile urinary tract infection rates after transrectal ultrasound-guided prostate biopsy compared with empiric prophylaxis; however, evidence remains insufficient to conclude an effect on the risk of sepsis.

## 1 Introduction

Ultrasound-guided prostate biopsy, via either the transrectal (transrectal ultrasound-guided prostate biopsy [TRUS-PB]) or the transperineal (transperineal ultrasound-guided prostate biopsy [TPUS-PB]) route, is the primary procedure for diagnosing prostate cancer (PCa) [1]. TPUS-PB allows omission of antibiotic prophylaxis while maintaining comparable detection and complication rates to TRUS-PB [2,3]. While some studies suggest that TPUS-PB seems to be associated with lower hospitalization and sepsis rates [4], others fail to confirm this [5]. The transrectal approach continues to be recommended and used widely, mainly owing to the complex technical demands and higher patient discomfort of TPUS-PB. The optimal antibiotic prophylaxis for patients planned for TRUS-PB remains; therefore, it is of utmost importance to minimize the risk of infectious complications secondary to the procedure.

Several methods have been investigated to reduce infection rates after TRUS-PB, including augmented prophylactic antibiotics, povidone-iodine rectal preparation, and targeted prophylactic antibiotics (TPAs) based on rectal swab or stool cultures. A previous meta-analysis [6] showed that the combination of prophylactic antibiotics and povidone-iodine rectal preparation before TRUS-PB reduces the incidence rate of febrile urinary tract infection (fUTI) significantly compared with empiric prophylactic antibiotic monotherapy (risk ratio [RR]: 0.47, 95% confidence interval [CI]: 0.3–0.75, *p* = 0.001). However, the effect of rectal swab–based TPAs remains unclear despite being studied in randomized controlled trials (RCTs) [[7], [8], [9]], likely due to the small sample size of each RCT. Therefore, there is a need for systematic data synthesis to ensure a pooling-based comprehensive evaluation.

We conducted this systematic review and meta-analysis of RCTs to investigate the efficacy of TPAs based on rectal swab cultures prior to TRUS-PB in reducing infectious complication rates compared with empirical antibiotic prophylaxis, focusing on the incidence of both fUTI and sepsis.

## 2 Methods

We registered the study with the International Prospective Register of Systematic Reviews (PROSPERO: registration number: CRD 42024523794). This systematic review and meta-analysis was conducted in line with the Preferred Reporting Items for Systematic Reviews and Meta-analyses (PRISMA) statement (PRISMA 2020 checklist; Supplementary Table 1).

### 2.1 Search strategy

In December 2024, the MEDLINE (via PubMed), Web of Science Core Collection, and Scopus databases were searched to identify studies investigating the effectiveness of TPAs based on rectal swab culture before TRUS-PB. The search terms included the following: “prostate biopsy,” “prophylactic antibiotics,” “randomized controlled trial,” and “prospective.” The detailed search strategy for each database is shown in the Supplementary material. In addition, searches of reference lists were performed to identify additional studies of interest. Two investigators independently performed an initial screening based on the titles and abstracts, and noted the cause of exclusion of ineligible reports. Full texts were retrieved and evaluated for eligibility. Discrepancies, if any, were solved by consensus among the authors.

### 2.2 Inclusion and exclusion criteria

We used the population, interventions, comparator, outcomes, and study design (PICOS) framework to define the eligibility criteria (Supplementary Table 2) [10]. We included studies that evaluated the efficacy of TPAs based on rectal swab culture prior to TRUS-PB for PCa detection (population and intervention). We compared these patients with those who were administered the standard empiric prophylactic antibiotics (control) to assess the incidence rate of infectious complications after TRUS-PB (outcomes). We included only RCTs (study design). We excluded studies that lacked original patient data, letters, editorial remarks, responses from authors, case reports, and non–English-language manuscripts. When encountering duplicate cohorts, we selected reports more relevant to our outcomes of interest.

### 2.3 Data extraction

Two reviewers independently extracted data on baseline study and patients’ characteristics. From each study, we retrieved the following data: first author’s name, publication year, country of origin, design of the study, timing of rectal swab culture, types of culture media, types and dosing of prophylactic antibiotics, study inclusion and exclusion criteria, primary endpoint, study group size, median age, diabetes mellitus (DM), estimated prostate volume (ePV), International Prostate Symptom Score (IPSS), postvoid residual urine volume (PVR), fUTI and sepsis definitions, incidence rate of fUTI, and incidence rate of sepsis. If the necessary outcomes were not directly available in the text, bar graphs were digitized using WebPlotDigitizer software (version 4.6) to retrieve relevant data [11,12]. All discrepancies were resolved by consensus with coauthors.

### 2.4 Quality assessment and risk of bias

Study quality and risk of bias were evaluated using the Risk-of-Bias (ROB version 2) tool, as outlined in the *Cochrane Handbook for Systematic Reviews of Interventions* [13]. The RoB2 assessment of each study was performed by two authors independently. Conflicts were resolved by consensus with coauthors.

### 2.5 Statistical analysis

All statistical analyses were performed using R Version 4.2.2 (2023, meta; R Foundation for Statistical Computing, Vienna, Austria). To evaluate the effectiveness of TPAs based on rectal swab culture prior to TRUS-PB regarding the incidence rate of fUTI or sepsis compared with empirical antibiotic prophylaxis, we pooled the RR and CI values for the incidence of fUTI and sepsis. Cochran’s Q test was used to evaluate the heterogeneity. When significant heterogeneity was observed, we attempted to investigate the causes of heterogeneity [14]. In addition, we calculated absolute event rates in each arm by pooling proportions using a random‑effect meta‑analysis with the Freeman-Tukey double‑arcsine transformation and the DerSimonian-Laird estimator via metaprop. The *p* values for the pooled RRs were calculated using the standard z test implemented in the R meta package. The pooled incidence in the empirical antibiotic group was defined as the control event rate (CER), and that in the TPA group was defined as the experimental event rate (EER). Absolute risk reduction (ARR) was calculated as “ARR = CER – EER,” and the number needed to treat (NNT) was calculated as “NNT = 1/ARR.” We preformed sensitivity analyses to increase homogeneity and confirm the reliability of our results. The publication bias was assessed using funnel plots. The *p* values of <0.05 were considered significant. All tests were two sided.

## 3 Results

### 3.1 Study selection and characteristics

**Fig. 1:**
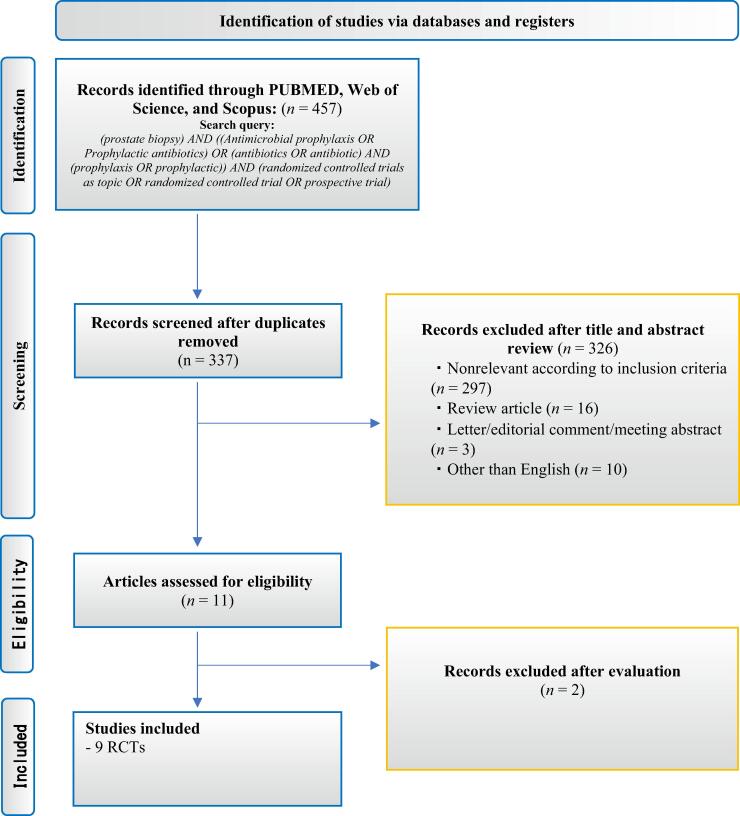
The Preferred Reporting Items for Systematic Reviews and Meta-analyses (PRISMA) flow chart, detailing the article selection process. RCT = randomized controlled trial.

A total of 457 records were initially identified through database search. After duplicate removal, 337 articles were screened, and 326 were excluded at the title and abstract level. Eleven full-text articles were assessed for eligibility, resulting in nine RCTs meeting the inclusion criteria. The search strategy is presented in Figure 1. According to our inclusion criteria, we identified nine RCTs [[7], [8], [9],[15], [16], [17], [18], [19], [20]] comprising 3002 patients eligible for meta-analyses. Seven studies [8,9,[15], [16], [17], [18], [19]] used ciprofloxacin as the empiric prophylactic antibiotic prior to TRUS-PB in the control groups. Five RCTs [8,9,15,18,19] used ciprofloxacin, and Sadahira et al [7] used levofloxacin as a first option for prophylactic antibiotics if quinolone-resistant microorganisms were not identified on the rectal swab, while the other three RCTs [16,17,20] used antibiotics based on rectal swab culture. Details of the administration of prophylactic antibiotics and the protocol of TPAs based on rectal swab are summarized in Supplementary Table 3. Six studies [[7], [8], [9],^16^,^18^,^19^] used selective screening media, while the others [15,17,20] used standard medium (Supplementary Table 4). Only one RCT, reported by Sadahira et al [7], described performing povidone-iodine disinfection before TRUS-PB, while two RCTs [8,9] reported that these did not perform povidone-iodine disinfection before TRUS-PB, and information on disinfection was not provided in the remaining studies. Regarding the patients’ characteristics, PVR was described in two studies [8,16], usage of antibiotics in the last 6 mo was described in three studies [8,16,18], and IPSS was investigated in two studies [8,9]. Details of other available patient characteristics are summarized in Table 1.

**Table 1:** Characteristics of included studies

Author (year) Country	Period	Intervention Control	No. of patients Total Intervention Control	Positive rate of QR bacteria, *n* (%)	Age (yr)	DM, *n* (%)	Prostate volume (ml) %	Postvoid residual urine volume (ml), %	IPSS	Usage of antibiotics in the last 6 mo, *n* (%)	Indwelling urinary catheter at biopsy, *n* (%)	Previous prostate biopsy, *n* (%)
Sadahira et al (2025) [7] Japan	2020–2024	I: TPA C: LVFX	373 I: 187 C: 186	67 (36)	I: 72 C: 73	0 (0)	>30 ml I: 111 (59%) C: 99 (53%)	Only including <100 ml	Only including <20	I: 4 (2.1) C: 1 (0.5)	NA	NA
Bouzouita et al (2024) [8] Tunisia	2016–2018	I: TPA C: CPFX	157 I: 80 C: 77	45 (56)	I: 65.1 C: 65.4 a	I: 27 (34) C: 23 (30)	I: 42.9 C: 62.9 a	I: 49.3 C: 45.8 a	IPSS moderate I: 58 (72%) C: 53 (69%)	I: 17 (21) C: 18 (23)	NA	I: 2 (2.5) C: 7 (9)
Tops et al (2023) [9] The Netherlands	2018–2021	I: TPA C: CPFX	1288 I: 652 C: 636	94 (15)	I: 69 C: 68	I: 71 (11) C: 49 (8) *p* < 0.05	I: 51 C: 50.7	NA	I: 9 C: 10 Median	NA	I: 12 (2) C: 22 (3)	I: 143 (23) C: 156 (24)
Benli et al (2022) [15] Turkey	2018–2020	I: TPA C: CPFX	140 I: 69 C: 71	1550 (30)	I: 65.3 C: 65.9 a	I: 15 (22) C: 15 (21)	NA	NA	NA	NA	NA	NA
Van Besien et al (2019) [16] Belgium	2015–2017	I: TPA C: CPFX	204 I: 102 C: 102	23 (23)	I: 64 C: 67 a	I: 12 (12) C: 8 (8)	>50 ml I: 48 (47%) C: 45 (44%)	>50 ml I: 11 (11%) C: 15 (15%)	NA	I: 17 (17) C: 24 (23)	NA	I: 17 (17) C: 16 (16)
Doherty et al (2019) [17] Nigeria	NA	I: TPA C: CPFX	100 I: 50 C: 50	29 (57)	66 a	I: 10 (20) C: 12 (24)	>100 ml I: 27 (54%) C: 23 (46%)	NA	NA	NA	NA	NA
Elshal et al (2018) [18] Egypt	2015–2017	I: TPA C: CPFX	330 I: 167 C: 163	139 (83.2)	I: 66 C: 66.4 a	I: 32 (19) C: 27 (17)	I: 75 C: 75	NA	NA	I: 14 (8) C: 20 (12)	I: 66 (40) C: 79 (48)	I: 4 (2.4) C: 2 (1.2)
Ozgur et al (2017) [19] Turkey	2012–2014	I: TPA C: CPFX	300 I: 144 C: 156	26 (18)	I: 63 C: 64	I: 36 (25) C: 32 (21)	I: 40 C: 46	NA	NA	NA	NA	NA
Kisa et al (2017) [20] Turkey	2014	I: TPA C: CPFX or FOM	110 b I: 32 C: 78	NA	63.8	16 (15)	NA	NA	NA	NA	NA	NA

C = control; CPFX = ciprofloxacin; DM = diabetes mellitus; FOM = fosfomycin; I = intervention; IPSS = International Prostate Symptom Score; LVFX = levofloxacin; NA = not applicable; QR = quinolone resistance; TPA = targeted prophylactic antibiotics based on rectal swab culture.

Values are presented as median unless otherwise indicated.

a Mean.

b Intervention group: groups B1 and B2; control group: groups A and B3.

### 3.2 Types, duration, and timing of prophylactic antibiotics in each study

The types, timing, and duration of both targeted and empiric prophylactic antibiotics varied among the included studies (Supplementary Table 3). While most empiric regimens consisted of single- or two-dose ciprofloxacin administered orally around the time of TRUS-PB, targeted prophylaxis protocols were more heterogeneous. These included intravenous or oral administration of antibiotics tailored to rectal swab culture results, with agents such as fosfomycin, cephalosporins, aminoglycosides, or beta-lactam/beta-lactamase inhibitors. Some studies applied multidose regimens or extended durations of prophylaxis, particularly in the targeted groups.

### 3.3 Definition of fUTI and sepsis in each study

The definitions of fUTI and sepsis varied across the included RCTs (Table 2). For fUTI, some studies [[7], [8], [9]] applied comprehensive criteria, including both systemic symptoms (eg, fever ≥38°C and malaise) and local urinary symptoms (eg, dysuria, urgency, and pyuria), while others only considered isolated fever or relied on clinical judgment. For sepsis, only three RCTs [7,9,18] clearly defined the diagnostic criteria; two used quick sequential organ failure assessment (qSOFA) [7,9], and one used the systemic inflammatory response syndrome (SIRS) criteria [18]. The remaining studies did not define sepsis explicitly [8,[15], [16], [17],^19^,^20^].

**Table 2:** Definition of febrile urinary tract infection and sepsis

Author (year) Country	fUTI	Sepsis
Sadahira et al (2025) [7] Japan	Pyuria and systemic symptoms such as a high fever over 38°C, urinary frequency or urgency, dysuria, and micturition pain without other infections	Based on qSOFA
Bouzouita et al (2024) [8] Tunisia	Prostatitis, genital infections, pyelonephritis, isolated fever >38°C with no extraurinary origin	NA
Tops et al (2023) [9] The Netherlands	Symptoms of dysuria, urgency, Frequency, or hematuria, and/or symptoms of fever, chills, or malaise and pyuria	Based on qSOFA
Benli et al (2022) [15] Turkey	High fever over 38.5°C	NA
Van Besien et al (2019) [16] Belgium	NA	NA
Doherty et al (2019) [17] Nigeria	Any body temperature >38°C	NA
Elshal et al (2018) [18] Egypt	Clinically suggested with positive urinalysis and culture	Based on SIRS criteria
Ozgur et al (2017) [19] Turkey	NA	NA
Kisa et al (2017) [20] Turkey	NA	NA

fUTI = febrile urinary tract infection; NA = not applicable; qSOFA = quick sequential organ failure assessment score; SIRS = systemic inflammatory response syndrome.

### 3.4 Risk of bias assessment

The authors’ judgments about each domain for each included study are illustrated in Supplementary Figure 1. Funnel plots of each analysis are depicted in Supplementary Figure 2.

### 3.5 Febrile urinary tract infections

**Fig. 2:**
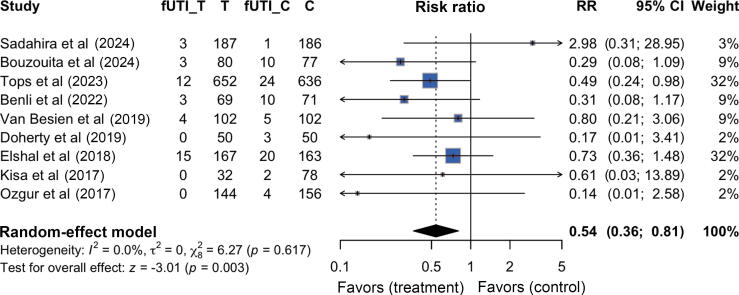
Forest plot of targeted prophylactic antibiotic group versus empiric antibiotic group regarding the incidence rate of febrile urinary tract infection. C = control group; CI = confidence interval; fUTI = febrile urinary tract infection; RR = risk ratio; T = targeted prophylactic antibiotic group.

Nine studies [[7], [8], [9],[15], [16], [17], [18], [19], [20]], comprising 3002 patients, reported the incidence rate of fUTI after TRUS-PB. The studies included 1483 patients who received TPAs and 1519 patients who received the empiric prophylactic antibiotics. Forty patients (2.7%) in the TPA group experienced an fUTI, compared with 79 (5.2%) in the empiric prophylactic antibiotic group. Patients who received TPAs based on rectal swab cultures had a significantly lower incidence of fUTI than those who received empiric prophylactic antibiotics (RR: 0.53, 95% CI: 0.35–0.78, *p* = 0.002; Fig. 2). The pooled CER was 5.2% and the pooled EER was 2.7%, yielding an ARR of 2.5 and an NNT of 40. We did not find sufficient evidence of a difference according to the Cochran’s Q tests. The funnel plot did not show any substantial asymmetry, suggesting a low risk of publication bias (Supplementary Fig. 2A).

### 3.6 Sepsis

**Fig. 3:**
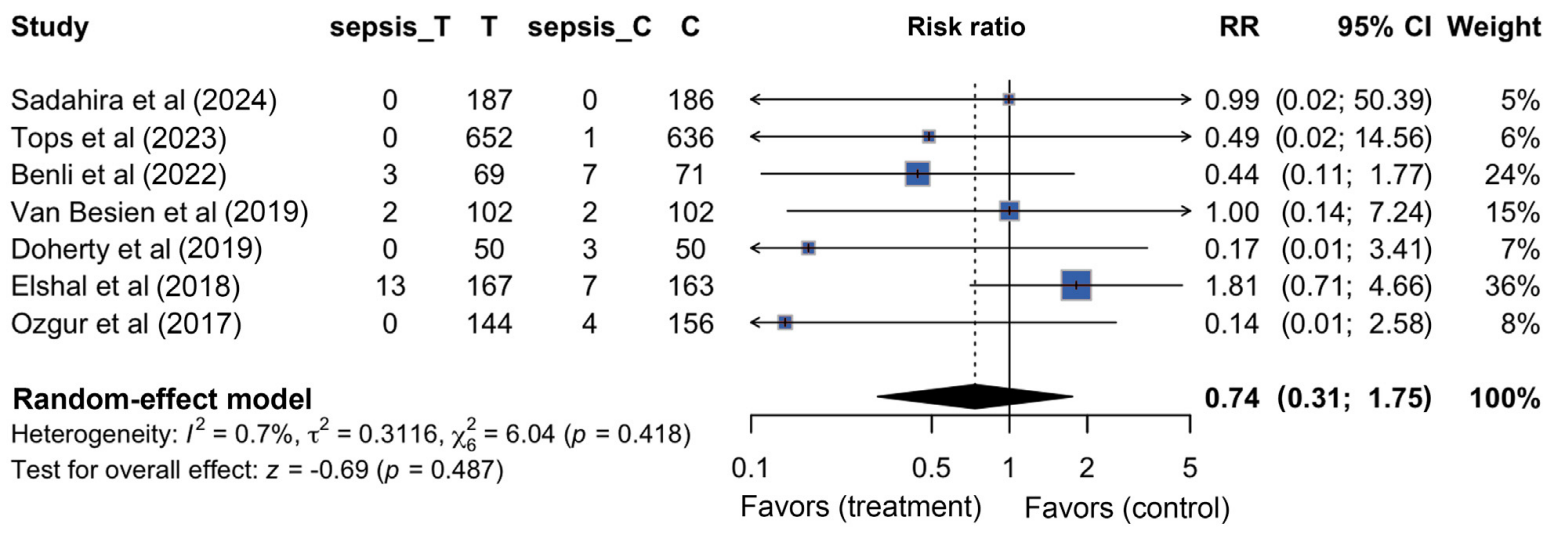
Forest plot of targeted prophylactic antibiotic groups versus empiric antibiotic group regarding the incidence rate of sepsis. C = control group; CI = confidence interval; RR = risk ratio; T = targeted prophylactic antibiotic group.

Seven studies [7,9,[15], [16], [17], [18], [19]], comprising 2735 patients, reported the incidence rate of sepsis after TRUS-PB with TPAs compared with empiric prophylactic antibiotics. The studies included 1371 patients in the TPA group and 1364 in the empiric prophylactic antibiotic group. Eighteen patients (1.3%) in the TPA group experienced sepsis compared with 24 (1.8%) in the empiric prophylactic antibiotic group. The difference between the two groups was not statistically significant (RR: 0.74, 95% CI: 0.31–1.75, *p* = 0.5; Fig. 3). We did not find sufficient evidence of a difference according to the Cochran’s Q tests. The funnel plot did not show any substantial asymmetry, suggesting a low risk of publication bias (Supplementary Fig. 2B).

## 4 Discussion

To the best of our knowledge, this is the first systematic review and meta-analysis of RCTs that analyzed the efficacy of TPAs based on rectal swab culture prior to TRUS-PB compared with empiric prophylactic antibiotics. We found that TPAs based on rectal swab culture reduced the risk of fUTI twofold compared with empiric prophylactic antibiotics; however, there was insufficient evidence to show a significant difference with regard to the risk of sepsis.

Although TPUS-PB is recommended in the current European Association of Urology (EAU) guidelines due to its lower infection rate, TRUS-guided biopsy is widely used across various clinical settings [3]. Over the past decades, the adoption of multiparametric magnetic resonance imaging (MRI) has improved the detection of clinically significant PCa, especially through MRI/ultrasound fusion-guided biopsy, which combines targeted and systematic sampling. However, this approach entails high costs and requires specific equipment and expertise, limiting its accessibility [21]. In contrast, TRUS-guided systematic biopsy or cognitive fusion targeted biopsy is still the primary method of prostate biopsy in many institutions owing to its low cost, procedural simplicity, short learning curve, and widespread availability [22]. A previous study based on an analysis of US private insurance claims revealed that the total cost of prostate biopsy was highest for MRI/ultrasound fusion biopsy ($4396), followed by TPUS ($2849), with TRUS ($1869) being the least costly [22]. Therefore, despite the theoretical advantages of TPUS-PB and MRI-guided techniques, TRUS-PB continues to play an important role in real-world practice.

We found that TPAs based on rectal swab culture reduced the risk of fUTI by 46% compared with empiric prophylactic antibiotics. Only one of the individual studies included in our analysis has shown that TPAs were significantly associated with a lower incidence rate of fUTI than empiric prophylactic antibiotics (RR: 0.49, 95% CI: 0.24–0.98) [9]. In the remaining RCTs, the difference between the two groups was not statistically significant, likely due to insufficient statistical power. However, each of these RCTs [8,[15], [16], [17], [18], [19]], except for one [7], reported that patients who received TPAs tended to have a lower incidence rate of fUTI, and the risk of serious infectious complications is particularly important considering the high incidence of PCa, and therefore, a high number of biopsies being performed. Sadahira et al [7] strictly excluded patients with a high risk of infectious complications after TRUS-PB, such as those with DM, ePV of >75 ml, severe lower urinary tract symptoms based on an IPSS of ≥20, maximum urinary flow rate of <12 ml/s, and/or PVR of >100 ml. Most of the included RCTs did not consider these risks of infectious complication after TRUS-PB. Elshal et al [18], on the contrary, included approximately 40% of patients with an indwelling urinary catheter at TRUS-PB, with a median ePV of 75 ml. Despite this heterogeneity in patient characteristics, we detected a statistically significant reduction of the fUTI rate after TRUS-PB when rectal swab–based TPAs was used compared with empiric prophylactic antibiotics. This highlights the importance of our meta-analysis and shows that TPAs based on rectal swab culture may benefit patients, in particular those at a high risk of infectious complications due to pre-existing conditions [7].

We found no significant difference in the incidence rate of sepsis after TRUS-PB between patients who received TPAs based on rectal swab culture and those who received empiric prophylactic antibiotics. However, the low incidence rate of sepsis after TRUS-PB in all studies may have limited the statistical power of our study. Therefore, further studies with larger sample sizes are needed to conclusively determine the efficacy of TPAs based on rectal swab cultures prior to TRUS-PB compared with empiric prophylactic antibiotics, in reducing the rate of sepsis. Although research focusing on TPAs for TRUS-guided biopsy may be considered somewhat outdated in terms of increasing preference for transperineal approaches, the potentially fatal nature of sepsis ensures that this topic remains of high clinical relevance.

In terms of cost effectiveness, we found that only Tops et al [9] indicated that the estimated additional cost of rectal swab culture was €79 per procedure. Our analyses revealed that the NNT to prevent one case of fUTI after TRUS-PB was 40. This calculation indicates that an additional cost of approximately €3200 is required to prevent one case of fUTI due to TRUS-PB. Although it is difficult to compare these costs with those required for treating fUTI, the implementation of TPUS-PB has been suggested to be more cost effective in reducing the incidence of fUTI. Indeed, a previous meta-analysis, including only RCTs, showed that TPUS-PB was associated with a significantly lower incidence rate of infectious complications than TRUS-PB (RR: 0.55, 95% CI: 0.33–0.92, *p* = 0.02) [23]. The EAU guidelines recommend TPUS-PB as the first choice [3]. However, in cases where TRUS-PB is planned, TPAs based on rectal swab culture should be considered to increase patients’ safety by the incidence rate of infectious complications.

Future trials should be powered adequately to assess low-incidence but high-impact events such as sepsis. In addition, our findings underscore the need for standardized definitions of infectious outcomes in future research. The adoption of uniform diagnostic criteria, such as Sepsis-3 guidelines–based definitions for UTI or sepsis, would improve comparability across studies and enhance the validity of meta-analytic estimates [24]. Evaluations of cost effectiveness and feasibility of targeted prophylaxis in real-world settings are also warranted to inform implementation strategies.

### 4.1 Limitations

There are several limitations to our study. Several studies failed to describe patients’ characteristics, including ePV, PVR, IPSS, and DM. In addition, data on baseline infection-related risk factors, such as indwelling urinary catheters and bacteriuria, were reported inconsistently. This lack of clinical detail limits the applicability of our findings, particularly to high-risk populations. Although we included only RCTs in our analyses, significant differences were observed in the baseline characteristics of patients among the included studies. Interestingly, despite the EAU guidelines not recommending fluoroquinolones as prophylactic antibiotics before TRUS-PB due to their adverse effect profile and the worldwide increase in quinolone-resistant microorganisms, most of the RCTs used fluoroquinolones either as empiric prophylactic antibiotics or when rectal swab culture were negative. Therefore, since the usage of prophylactic antibiotics is anticipated to change moving forward, these findings cannot be extrapolated directly to current and future clinical practice. Moreover, although the prevalence of quinolone-resistant bacteria varied among the included studies, this factor was not considered in our study. Therefore, the influence of quinolone-resistant bacteria on the effectiveness of TPAs based on rectal swab in reducing the incidence of fUTI remains unclear. Although the EAU guidelines recommend povidone-iodine rectal preparation prior to TRUS-PB, some of the included studies did not perform it and others did not describe whether it was performed or not. We believed that this difference contributed to significant heterogeneity among the included studies. In addition, the types, duration, and timing of TPAs and empiric prophylactic antibiotics varied among the RCTs (Supplementary Table 3); the timing of rectal swab culture and the type of culture medium also varied among the RCTs (Supplementary Table 4). One of the limitations of our analysis is the heterogeneity in definitions of fUTI and sepsis across the included RCTs. Only a subset of studies reported explicit diagnostic criteria for these outcomes. For sepsis, in particular, the use of different criteria, such as qSOFA and SIRS, or a lack of definitions may have introduced a classification bias. This inconsistency likely reduced the precision of our pooled estimates, especially for sepsis, which was a relatively rare event.

## 5 Conclusions

We found that TPAs based on a rectal swab culture led to a significant decrease in the rate of fUTI after TRUS-PB compared with empiric prophylactic antibiotics. However, there was insufficient evidence to detect a significant difference in the rate of sepsis between the two approaches. Based on the clinically relevant reduction in the incidence of fUTI, we recommend performing rectal swab–based TPAs in patients undergoing TRUS-PB.

  ***Author contributions*:** Shahrokh F. Shariat had full access to all the data in the study and takes responsibility for the integrity of the data and the accuracy of the data analysis.

  *Study concept and design*: Tsuboi, Parizi.

*Acquisition of data*: Tsuboi.

*Analysis and interpretation of data*: Tsuboi.

*Drafting of the manuscript*: Tsuboi, Parizi.

*Critical revision of the manuscript for important intellectual content*: Matsukawa, Miszczyk, Fazekas, Cormio, Kawada, Katayama, Iwata, Bekku, Wada, Karakiewicz, Chlosta, Briganti.

*Statistical analysis*: Tsuboi.

*Obtaining funding*: None.

*Administrative, technical, or material support*: None.

*Supervision*: Araki, Shariat.

*Other*: None.

  ***Financial disclosures:*** Shahrokh F. Shariat certifies that all conflicts of interest, including specific financial interests and relationships and affiliations relevant to the subject matter or materials discussed in the manuscript (eg, employment/affiliation, grants or funding, consultancies, honoraria, stock ownership or options, expert testimony, royalties, or patents filed, received, or pending), are the following: Shahrokh F. Shariat received honoraria from Astellas, AstraZeneca, BMS, Ferring, Ipsen, Janssen, MSD, Olympus, Pfizer, Roche, and Takeda; reports consulting or advisory role at Astellas, AstraZeneca, BMS, Ferring, Ipsen, Janssen, MSD, Olympus, Pfizer, Pierre Fabre, Roche, and Takeda; and reports being a speakers’ bureau member for Astellas, Astra Zeneca, Bayer, BMS, Ferring, Ipsen, Janssen, MSD, Olympus, Pfizer, Richard Wolf, Roche, and Takeda.

  ***Funding/Support and role of the sponsor*:** None.

  ***Acknowledgments*:** Tamás Fazekas was supported by the EUSP Scholarship of the 10.13039/501100003083European Association of Urology (Scholarship S-2023-0006). Marcin Miszczyk was supported by 10.13039/501100014434NAWA – Polish National Agency for Academic Exchange in cooperation with Medical Research Agency under the Walczak Programme, grant number BPN/WAL/2023/1/00061/DEC/1.
