# Supplementary material for: Rectal Swab–based Targeted Prophylactic Antibiotics Reduce Infectious Complications After Transrectal Prostate Biopsy: A Systematic Review and Meta-analysis of Randomized Controlled Trials
Source: Eur Urol Open Sci. 2025 Sep 11;80:57–65. doi: 10.1016/j.euros.2025.08.007 (PMC12501354; doi:10.1016/j.euros.2025.08.007)

**Supplementary Information**

**Supplementary Appendix 1**. Search strategy for meta-analysis

**Supplementary Table 1.** PRISMA checklist 2020

**Supplementary Table 2.** PICOS

**Supplementary Table 3.** Types, duration, and timing of prophylactic antibiotics of each study

**Supplementary Table 4.** Timing of rectal swab culture and type of medium of each study

**Supplementary Figure 1.** Risk of bias assessment of the included RCTs

**Supplementary Figure 2.** Funnel Plot

**Abbreviations:**

ARR = absolute risk reduction

CER = control event rate

CI = confidence interval

DM = diabetes mellites

EAU = European Association of Urology

EER = experimental event rate

ePV = estimated prostate volume

fUTI = febrile urinary tract infection

IPSS = international prostate symptom score

NNT = number needed to treat

PCa = prostate cancer

PRISMA = preferred reporting items for systematic reviews and meta-analyses

PVR = post-void residual urine volume

RCTs = randomized controlled trials

ROB2 = risk of bias version 2

RR = risk ratios

TPA = targeted prophylactic antibiotics

TPUS-PB = Tranperineal ultrasound-guided prostate biopsy

TRUS-PB = Tranrectal ultrasound-guided prostate biopsy

**Supplementary Appendix 1**. Search strategy for systematic review and meta-analysis

Electronic searches were performed in the following databases to identify eligible studies.

1. PubMed (n = 218)
2. SCOPUS (n = 12)
3. Web of science (n = 227)

There were no language or publication period limitations.

**PubMed**

1. Search: “prostate” [Title/Abstract]
2. Search: “biopsy” [Title/Abstract]
3. Search: “Antimicrobial prophylaxis” [Title/Abstract]
4. Search: “Prophylactic antibiotics” [Title/Abstract]
5. Search: “antibiotic*” [Title/Abstract]
6. Search: “prophylaxis” [Title/Abstract]
7. Search: “prophylactic” [Title/Abstract]
8. Search: “randomized controlled trials as topic” [MeSH Terms]
9. Search: “randomized controlled trial” [Publication Type]
10. Search: “randomized controlled trial” [Title/Abstract]
11. Search: “prospective*” [Title/Abstract]
12. #1 AND #2
13. #3 OR #4 OR #5 OR #6 OR #7
14. #8 OR#9 OR #10 OR #11
15. #12 AND #13 AND #14

**SCOPUS**

TITLE-ABS("prostate") AND TITLE-ABS("biopsy") AND (TITLE-ABS("Antimicrobial prophylaxis") OR TITLE-ABS("Prophylactic antibiotics") OR TITLE-ABS("antibiotic*") OR TITLE-ABS("prophylaxis") OR TITLE-ABS("prophylactic")) AND (TITLE-ABS("randomized controlled trial") OR PUBYEAR AFT 2000 AND TITLE-ABS("prospective*") OR PUBYEAR AFT 2000 AND "randomized controlled trials as topic")

**Web of science**

(TS=("prostate") AND TS=("biopsy") AND (TS=("Antimicrobial prophylaxis") OR TS=("Prophylactic antibiotics") OR TS=("antibiotic*") OR TS=("prophylaxis") OR TS=("prophylactic")) AND (TS=("randomized controlled trial") OR TS=("prospective*") OR TS=("randomized controlled trials as topic")))

**Supplementary Table 1.** PRISMA 2020 for Abstracts Checklist and PRISMA checklist 2020

| **Section and Topic** | **Item #** | **Checklist item** | | | | **Reported (Yes/No)** | |
| --- | --- | --- | --- | --- | --- | --- | --- |
| **TITLE** | | | | |  | |  |
| Title | | | 1 | Identify the report as a systematic review. | Yes | |  |
| **BACKGROUND** | | | | |  | |  |
| Objectives | | | 2 | Provide an explicit statement of the main objective(s) or question(s) the review addresses. | Yes | |  |
| **METHODS** | | | | |  | |  |
| Eligibility criteria | | | 3 | Specify the inclusion and exclusion criteria for the review. | No | |  |
| Information sources | | | 4 | Specify the information sources (e.g. databases, registers) used to identify studies and the date when each was last searched. | Yes | |  |
| Risk of bias | | | 5 | Specify the methods used to assess risk of bias in the included studies. | No | |  |
| Synthesis of results | | | 6 | Specify the methods used to present and synthesise results. | Yes | |  |
| **RESULTS** | | | | |  | |  |
| Included studies | | | 7 | Give the total number of included studies and participants and summarise relevant characteristics of studies. | Yes | |  |
| Synthesis of results | | | 8 | Present results for main outcomes, preferably indicating the number of included studies and participants for each. If meta-analysis was done, report the summary estimate and confidence/credible interval. If comparing groups, indicate the direction of the effect (i.e. which group is favoured). | Yes | |  |
| **DISCUSSION** | | | | |  | |  |
| Limitations of evidence | | | 9 | Provide a brief summary of the limitations of the evidence included in the review (e.g. study risk of bias, inconsistency and imprecision). | Yes | |  |
| Interpretation | | | 10 | Provide a general interpretation of the results and important implications. | Yes | |  |
| **OTHER** | | | | |  | |  |
| Funding | | | 11 | Specify the primary source of funding for the review. | No | |  |
| Registration | | | 12 | Provide the register name and registration number. | Yes | |  |
| **Section and Topic** | **Item #** | **Checklist item** | | | | **Location where item is reported** | |
| **TITLE** | | | | | |  | |
| Title | 1 | Identify the report as a systematic review. | | | | 1 | |
| **ABSTRACT** | | | | | |  | |
| Abstract | 2 | See the PRISMA 2020 for Abstracts checklist. | | | | 3-4 | |
| **INTRODUCTION** | | | | | |  | |
| Rationale | 3 | Describe the rationale for the review in the context of existing knowledge. | | | | 5 | |
| Objectives | 4 | Provide an explicit statement of the objective(s) or question(s) the review addresses. | | | | 5 | |
| **METHODS** | | | | | |  | |
| Eligibility criteria | 5 | Specify the inclusion and exclusion criteria for the review and how studies were grouped for the syntheses. | | | | 6-7 | |
| Information sources | 6 | Specify all databases, registers, websites, organisations, reference lists and other sources searched or consulted to identify studies. Specify the date when each source was last searched or consulted. | | | | 6-7 | |
| Search strategy | 7 | Present the full search strategies for all databases, registers and websites, including any filters and limits used. | | | | 6-7 | |
| Selection process | 8 | Specify the methods used to decide whether a study met the inclusion criteria of the review, including how many reviewers screened each record and each report retrieved, whether they worked independently, and if applicable, details of automation tools used in the process. | | | | 6-7 | |
| Data collection process | 9 | Specify the methods used to collect data from reports, including how many reviewers collected data from each report, whether they worked independently, any processes for obtaining or confirming data from study investigators, and if applicable, details of automation tools used in the process. | | | | 6-7 | |
| Data items | 10a | List and define all outcomes for which data were sought. Specify whether all results that were compatible with each outcome domain in each study were sought (e.g. for all measures, time points, analyses), and if not, the methods used to decide which results to collect. | | | | 6-7 | |
|  | 10b | List and define all other variables for which data were sought (e.g. participant and intervention characteristics, funding sources). Describe any assumptions made about any missing or unclear information. | | | | 6-7 | |
| Study risk of bias assessment | 11 | Specify the methods used to assess risk of bias in the included studies, including details of the tool(s) used, how many reviewers assessed each study and whether they worked independently, and if applicable, details of automation tools used in the process. | | | | 6-7 | |
| Effect measures | 12 | Specify for each outcome the effect measure(s) (e.g. risk ratio, mean difference) used in the synthesis or presentation of results. | | | | 6-7 | |
| Synthesis methods | 13a | Describe the processes used to decide which studies were eligible for each synthesis (e.g. tabulating the study intervention characteristics and comparing against the planned groups for each synthesis (item #5)). | | | | 6-7 | |
|  | 13b | Describe any methods required to prepare the data for presentation or synthesis, such as handling of missing summary statistics, or data conversions. | | | | 6-7 | |
|  | 13c | Describe any methods used to tabulate or visually display results of individual studies and syntheses. | | | | 6-7 | |
|  | 13d | Describe any methods used to synthesize results and provide a rationale for the choice(s). If meta-analysis was performed, describe the model(s), method(s) to identify the presence and extent of statistical heterogeneity, and software package(s) used. | | | | 6-7 | |
|  | 13e | Describe any methods used to explore possible causes of heterogeneity among study results (e.g. subgroup analysis, meta-regression). | | | | 6-7 | |
|  | 13f | Describe any sensitivity analyses conducted to assess robustness of the synthesized results. | | | | 6-7 | |
| Reporting bias assessment | 14 | Describe any methods used to assess risk of bias due to missing results in a synthesis (arising from reporting biases). | | | | 6-7 | |
| Certainty assessment | 15 | Describe any methods used to assess certainty (or confidence) in the body of evidence for an outcome. | | | | 6-7 | |
| **RESULTS** | | | | | |  | |
| Study selection | 16a | Describe the results of the search and selection process, from the number of records identified in the search to the number of studies included in the review, ideally using a flow diagram. | | | | 7-9 | |
|  | 16b | Cite studies that might appear to meet the inclusion criteria, but which were excluded, and explain why they were excluded. | | | | 7-9 | |
| Study characteristics | 17 | Cite each included study and present its characteristics. | | | | 7-9 | |
| Risk of bias in studies | 18 | Present assessments of risk of bias for each included study. | | | | 7-9 | |
| Results of individual studies | 19 | For all outcomes, present, for each study: (a) summary statistics for each group (where appropriate) and (b) an effect estimate and its precision (e.g. confidence/credible interval), ideally using structured tables or plots. | | | | 7-9 | |
| Results of syntheses | 20a | For each synthesis, briefly summarise the characteristics and risk of bias among contributing studies. | | | | 7-9 | |
|  | 20b | Present results of all statistical syntheses conducted. If meta-analysis was done, present for each the summary estimate and its precision (e.g. confidence/credible interval) and measures of statistical heterogeneity. If comparing groups, describe the direction of the effect. | | | | 7-9 | |
|  | 20c | Present results of all investigations of possible causes of heterogeneity among study results. | | | | 7-9 | |
|  | 20d | Present results of all sensitivity analyses conducted to assess the robustness of the synthesized results. | | | | 7-9 | |
| Reporting biases | 21 | Present assessments of risk of bias due to missing results (arising from reporting biases) for each synthesis assessed. | | | | 7-9 | |
| Certainty of evidence | 22 | Present assessments of certainty (or confidence) in the body of evidence for each outcome assessed. | | | | 7-9 | |
| **DISCUSSION** | | | | | |  | |
| Discussion | 23a | Provide a general interpretation of the results in the context of other evidence. | | | | 9-11 | |
|  | 23b | Discuss any limitations of the evidence included in the review. | | | | 9-11 | |
|  | 23c | Discuss any limitations of the review processes used. | | | | 9-11 | |
|  | 23d | Discuss implications of the results for practice, policy, and future research. | | | | 9-11 | |
| **OTHER INFORMATION** | | | | | |  | |
| Registration and protocol | 24a | Provide registration information for the review, including register name and registration number, or state that the review was not registered. | | | | 5 | |
|  | 24b | Indicate where the review protocol can be accessed, or state that a protocol was not prepared. | | | | NA | |
|  | 24c | Describe and explain any amendments to information provided at registration or in the protocol. | | | | NA | |
| Support | 25 | Describe sources of financial or non-financial support for the review, and the role of the funders or sponsors in the review. | | | | Authorship form | |
| Competing interests | 26 | Declare any competing interests of review authors. | | | | Authorship form | |
| Availability of data, code and other materials | 27 | Report which of the following are publicly available and where they can be found: template data collection forms; data extracted from included studies; data used for all analyses; analytic code; any other materials used in the review. | | | | NA | |

**Supplementary Table 2**: Study design according to the Population, Intervention, Control, Outcome, Study Design (PICOS) method

| Population | Male patients who underwent transrectal prostate biopsy for cancer screening or diagnosis |
| --- | --- |
| Intervention | Targeted prophylactic antibiotic therapy based on rectal swab culture |
| Control | Standard or empiric prophylactic antibiotic therapy |
| Outcome | Rate of post-biopsy infectious complications (fever UTI, sepsis) |
| Study design | randomized controlled trials |

**Supplementary Table 3**: Types, duration, and timing of prophylactic antibiotics of each study

| Author | Types, duration, and timing of targeted prophylactic antibiotics | Types, duration, and timing of empiric prophylactic antibiotics |
| --- | --- | --- |
| Sadahira et al. 2024 | ・Patients in the culture-negative group received on dose of LVFX 500mg orally two hours before TRUS-PB  ・Patients in the culture-positive group received TAZ/PIPC 4.5g 30min before TRPB and four hours after TRPB | ・Patients in the non-culture group received on dose of LVFX 500mg orally two hours before TRUS-PB |
| Bouzouita et al. 2024 | If FQ-resistant organisms were identified on the rectal swab test, prophylactic antibiotics was given as a first choice with third-generation cephalosporin or trimethoprim/sulfamethoxazole in the case of sensitivity to these molecules. Otherwise, aminoglycosides, piperacillin–tazobactamin, and imipenem were suggested. If no FQ-resistant organisms were identified, a single dose of oral Ciprofloxacin 500 mg was administered 60 min before TRUS-PB | Oral ciprofloxacin 500 mg was administered 60 min before TRUS-PB |
| Tops et al. 2023 | ・Patients who did not have ciprofloxacin resistance, based on the result of rectal swab culture, received ciprofloxacin 500mg orally two hours and 12 hours after TRUS-PB  ・Patients with ciprofloxacin resistance received the most appropriate prophylactic antibiotic according to the study’s protocol  Ciprofloxacin (86.2%), Fosfomycin (3.5%), Ciprofloxacin+ trimethoprim/sulfamethoxazole (3.1%) | Oral ciprofloxacin 500 mg was administered two hours before and 12 hours after TRUS-PB |
| Benli et al. 2022 | Targeted antibiotic prophylaxis based on the rectal swab culture sensitivity  Ceftriaxone (37.7%), Gentamicin (21.7%), ciprofloxiacin (21.7%), Amikacin (15.9%), Cefrazidime (2.9%) | Oral ciprofloxacin was administered two hours before and 12 hours after TRUS-PB |
| Van Besien et al. 2019 | In the case of growth of ciprofloxacin resistant organisms, patients in the intervention group were advised by a telephone call to take fosfomycin 3 g 2 h before the biopsy instead of ciprofloxacin. | Oral ciprofloxacin 500 mg was administered two hours before TRUS-PB |
| Doherty et al. 2019 | The interventions group had intravenous antibiotics based on the rectal swab culture sensitivity. | Intravenous ciprofloxacin 200 mg within 1 h before TRUS-PB |
| Elshal et al. 2018 | antibiotic was given just before TRUS-PB according to rectal swab culture. | Oral ciprofloxacin 500 mg for 3 days starting the day before TRUS-PB |
| Ozgur et al. 2017 | ・Patients who did not have ciprofloxacin resistance, based on the result of rectal swab culture, received ciprofloxacin prophylaxis for 3 days  ・Patients with ciprofloxacin resistance and/or ESBL production received the most appropriate prophylactic antibiotic based on the antibiogram. Drugs that have oral forms were given for prophylaxis, 3 days like ciprofloxacin and the others were given only 1 hour before the procedure with parenteral ways | Oral ciprofloxacin 500 mg twice a day for 3 days since the day before TRUS-PB |
| Kisa et al. 2017 | Patients was administered fosfomycin or oral ciprofloxacin based on the rectal swab culture | ・single dose Fosfomycin the night before TRUS-PB  ・those using ciprofloxacin twice daily for 5 days, beginning before TRUS-PB |

**Supplementary Table 4.** Timing of rectal swab culture and type of medium of each study

| Author | Timing of rectal swab | Type of medium |
| --- | --- | --- |
| Sadahira et al. 2024 | N/A | two kinds of CHROMagarTM media, developed to detect FQ-resistant, ESBL-producing *E. coli* (Kanto Chemical Co., Inc., Tokyo, Japan). |
| Bouzouita et al. 2024 | 10 days before TRUS-PB | desoxycholate lactose agar plates supplemented with ciprofloxacin (0.05 μg/ml) as selective screening agar for the isolation of Gram-negative bacilli resistant to FQs. |
| Tops et al. 2023 | At home by self-sampling approximately 14 days before TRUS-PB | MacConkey medium with vancomycin for isolating gram-negative bacilli, with added antibiotics: ciprofloxacin (0.5 mg/L), trimethoprim (2 mg/L), fosfomycin (4 mg/L) with glucose-6-phosphate (25 mg/L), and mecillinam (2 mg/L) with amoxicillin/clavulanic acid (8 mg/L) |
| Benli et al. 2022 | N/A | 5% sheep blood agar and eosin methylene blue agar (both from Salubris, Istanbul, Turkey) |
| Van Besien et al. 2019 | 3 - 30 days before TRUS-PB | MacConkey agar with ciprofloxacin 1 mg/L |
| Doherty et al. 2019 | 5 days before TRUS-PB in the clinic | MacConkey agar |
| Elshal et al. 2018 | 14 days before TRUS-PB | MacConkey agar with ciprofloxacin 10 μg/ml |
| Ozgur et al. 2017 | 7 - 14 days before TRUS-PB | MacConkey agar with ciprofloxacin (concentration unknown) |
| Kisa et al. 2017 | 3 - 7 days before TRUS-PB and on admission | N/A |

**Supplementary Figure 1.** Risk of bias assessment of the included RCTs


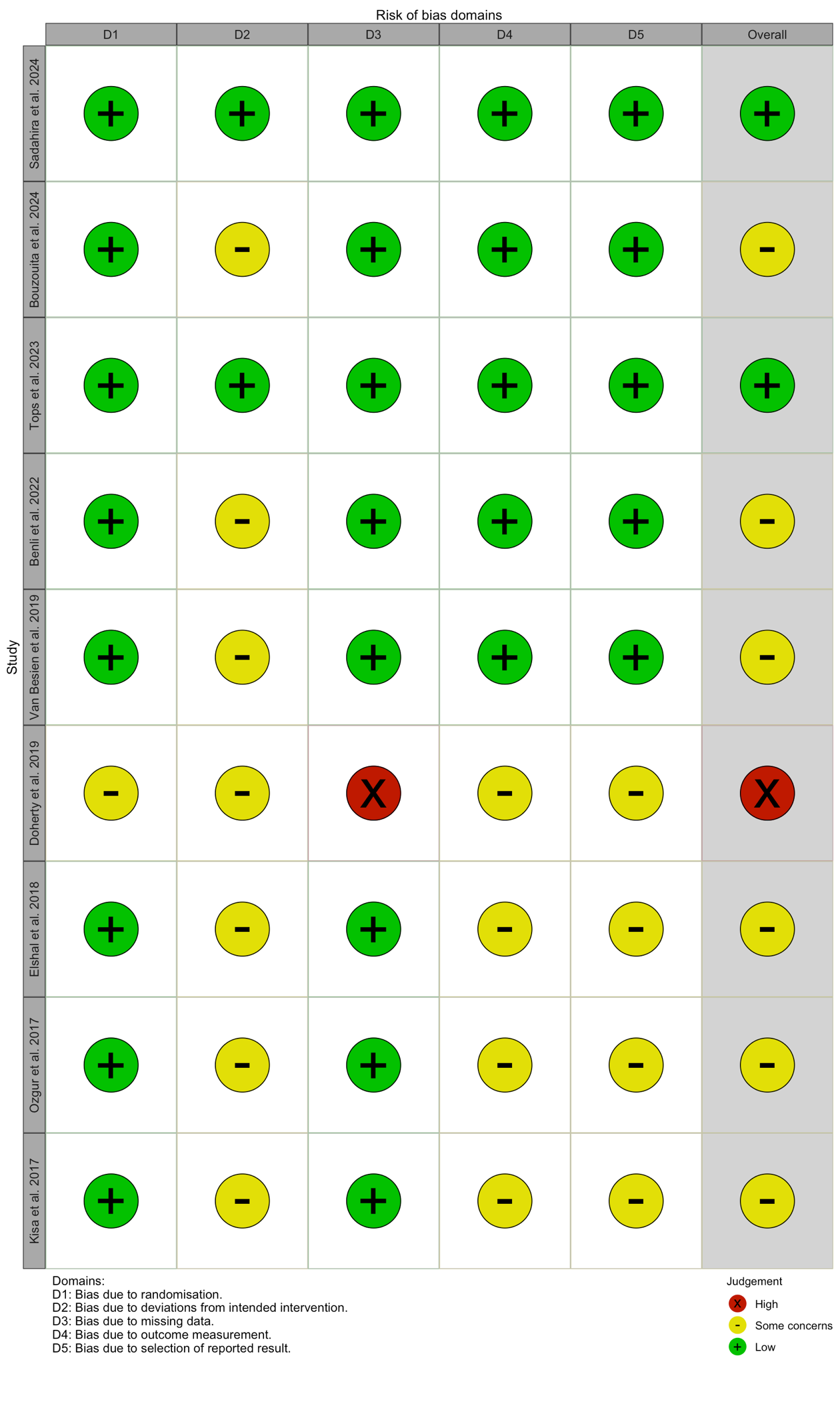


**Supplementary Figure 2.** Funnel Plot

1. Febrile UTI

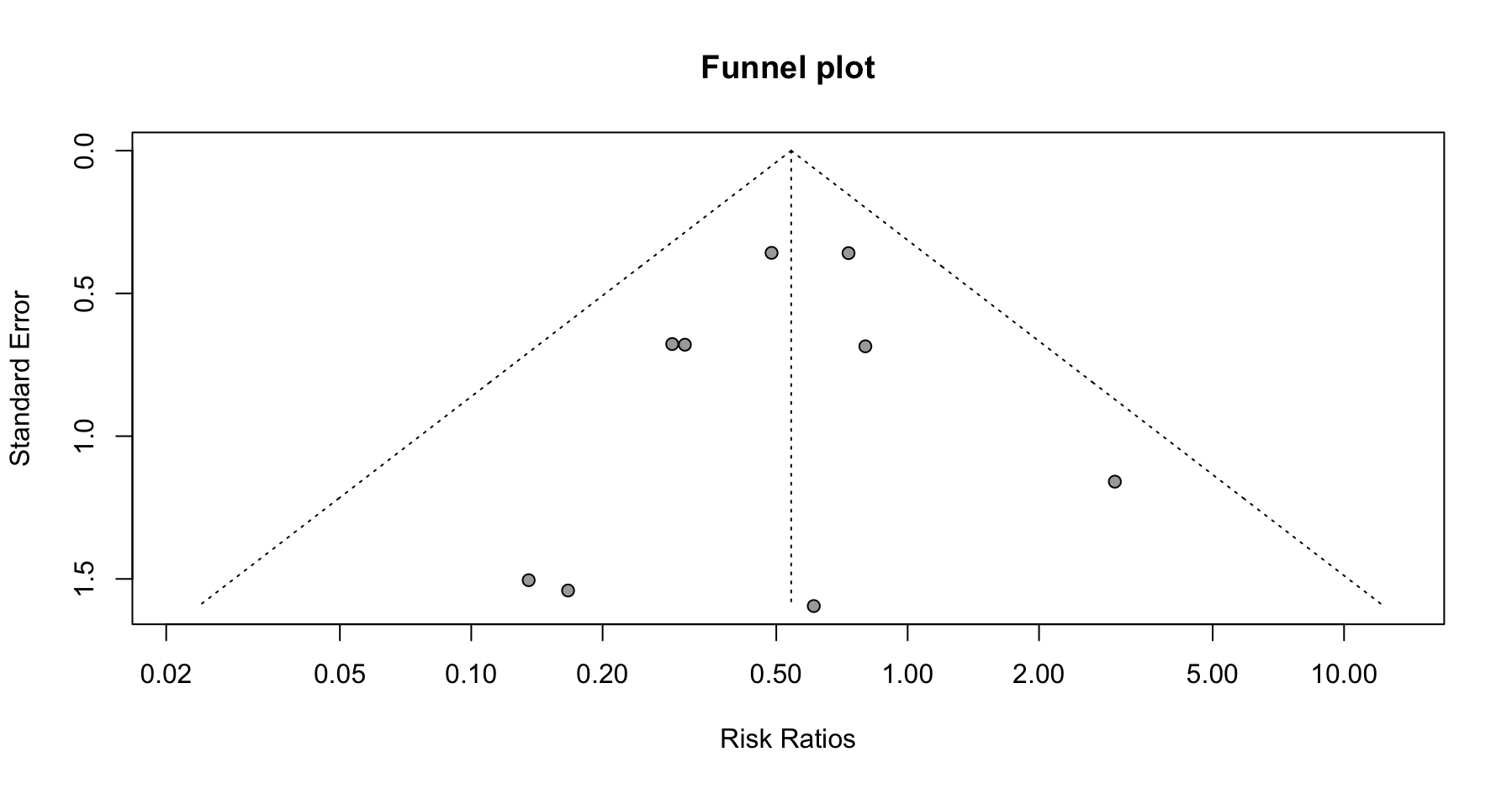

2. Sepsis

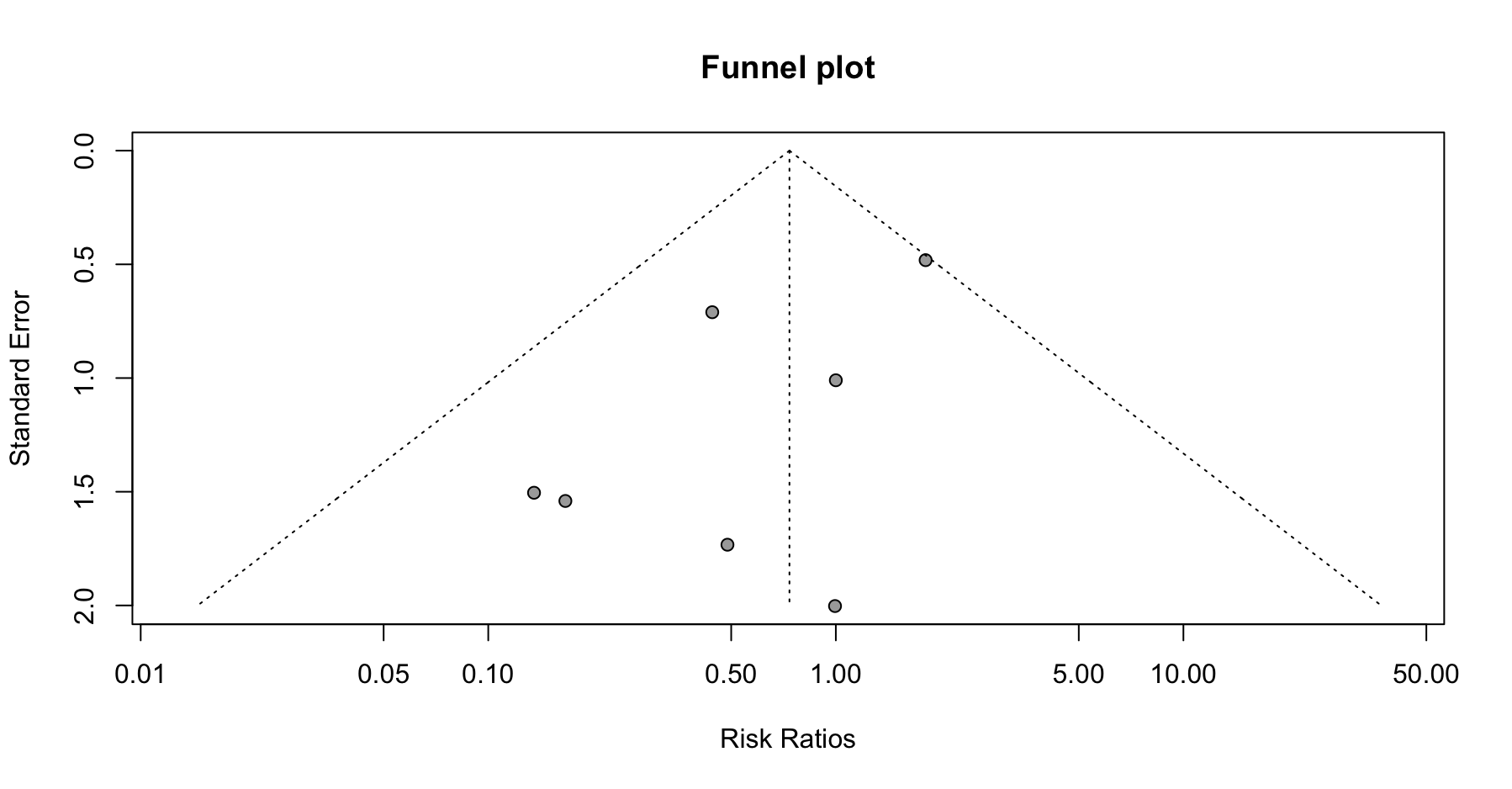

Supplement: Supplementary Data 1 [file mmc1.docx]
